# Forward-viewing echoendoscope-guided pancreaticojejunostomy for post-pancreaticoduodenectomy stricture

**DOI:** 10.1055/a-2589-0654

**Published:** 2025-05-09

**Authors:** Toru Kaneko, Mitsuhiro Kida, Takahiro Kurosu, Yutaro Saito, Shiori Koyama, Tomohiro Betto, Chika Kusano

**Affiliations:** 1 214303 Department of Gastroenterology, Kitasato University Medical Center, Kitamoto, Japan; 2 73444 Department of Gastroenterology, Kitasato University Hospital, Minami Sagamihara, Japan

Pancreaticojejunal anastomotic strictures (PJAS) and pancreatic fluid leakage can occur after pancreaticoduodenectomy 1 2 . Treatments include endoscopic retrograde pancreatography using a balloon enteroscope or transgastric endoscopic ultrasound (EUS)-guided procedures 2 3 . These treatments can be challenging in patients with severe or complete anastomotic obstruction. Alternatively, EUS-guided pancreaticojejunostomy (EUS-PJS) allows direct access to the pancreatic duct 4 , and a forward-viewing echoendoscope (FV-EUS; TGF-UCT260J; Olympus Medical Systems) expands the field of view, facilitating the precise identification of and access to the anastomotic site 4 . Herein, we describe our experience implementing EUS-PJS.

A 76-year-old man presented with PJAS and pancreatic fluid leakage 8 months after pancreaticoduodenectomy ( Fig. 1 ). Endoscopic treatment was planned, but single-balloon enteroscopy could not identify the pancreaticojejunostomy site. An EUS-guided rendezvous technique ( Fig. 2 **a** ) was attempted, but neither a guidewire (GW) nor contrast medium could pass through the pancreaticojejunostomy site; both advanced into the pancreatic fluid leakage area ( Fig. 2 **b, c** ). Transgastric EUS-guided drainage was performed for pancreatic fluid leakage on the same day ( Fig. 2 **d** ). Subsequently, EUS-PJS was performed using FV-EUS ( Fig. 3 , Video 1 ), which was advanced to the pancreaticojejunostomy site. The pancreatic duct was identified using EUS, punctured with a 19 G needle (EZ shot3; Olympus Medical Systems), and confirmed with contrast medium ( Fig. 4 **a** ), then a 0.025-inch GW was inserted ( Fig. 4 **b** ). The double-GW technique was employed due to significant angulation of the pancreatic duct, and dilation was performed using a drill-type dilator ( Fig. 4 **c** ). A double-lumen catheter was inserted while retaining 0.035 inches of GW in the pancreatic duct ( Fig. 4 **d** ). A 3-mm balloon dilator was used to dilate the pancreaticojejunostomy site ( Fig. 4 **e** ). A 7Fr 5-cm plastic stent was placed to complete the procedure ( Fig. 4 **f** ). Postoperative adverse events did not occur. EUS-PJS can treat PJAS if balloon enteroscopy or a transgastric EUS-guided approach is unsuccessful.

**Fig. 1:**
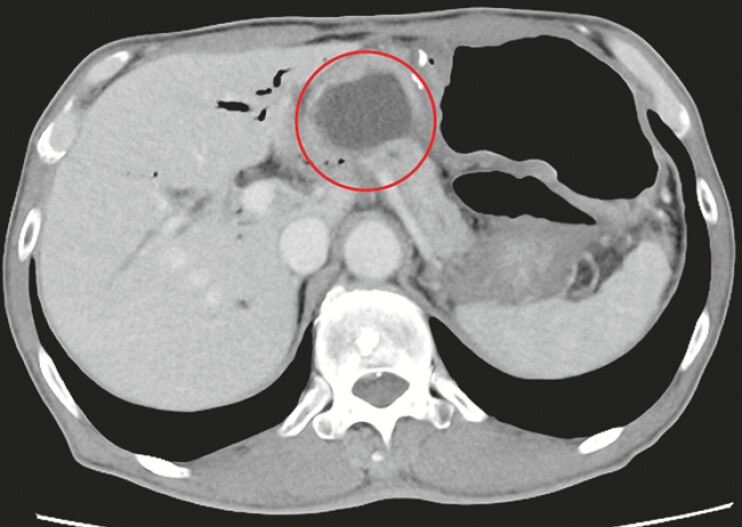
Pancreatic fistula (red circle) near the pancreaticojejunostomy site.

**Fig. 2:**
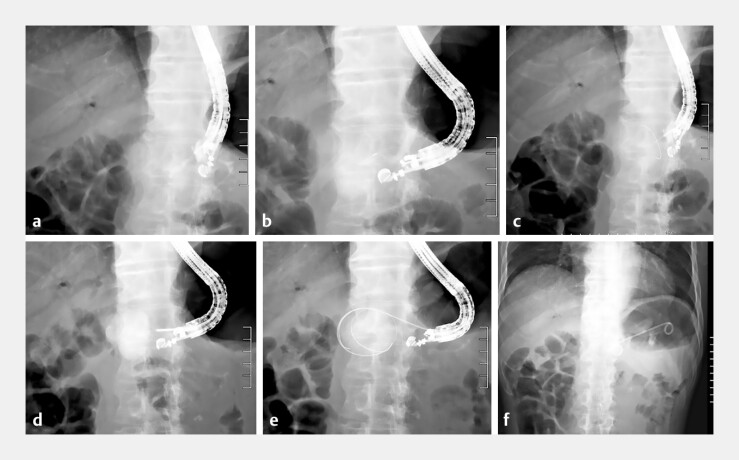
Endoscopic ultrasound-guided rendezvous technique and EUS-guided pancreatic fistula drainage. **a** Pancreatic duct puncture via the stomach and contrast medium injection. **b** Only the pancreatic fistula is visualized from the pancreatic duct. **c** Guidewire advancement stops at the pancreaticojejunostomy site and advances only to the pancreatic fistula. **d** Puncture of and contrast medium injection into the pancreatic fistula via transgastric using EUS. **e** Guidewire placement in the pancreatic fistula. **f** Transgastric stent placement in the pancreatic fistula. Abbreviation: EUS, endoscopic ultrasound.

**Fig. 3:**
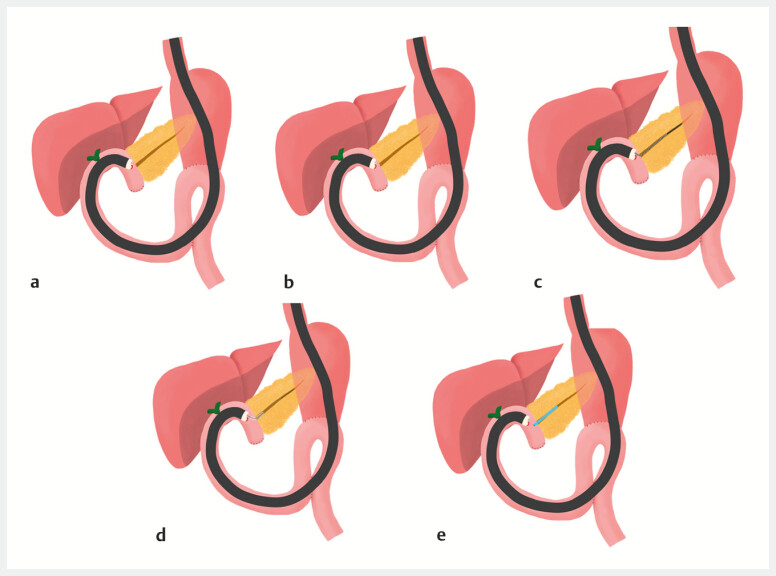
Schema of EUS-guided pancreaticojejunostomy. **a** Insertion of a forward-viewing echoendoscope into the pancreaticojejunostomy site. **b** Pancreatic duct puncture. **c** Guidewire placement in the pancreatic duct. **d** Puncture site dilation. **e** Stent placement in the pancreatic duct. Abbreviation: EUS, endoscopic ultrasound.

**Fig. 4:**
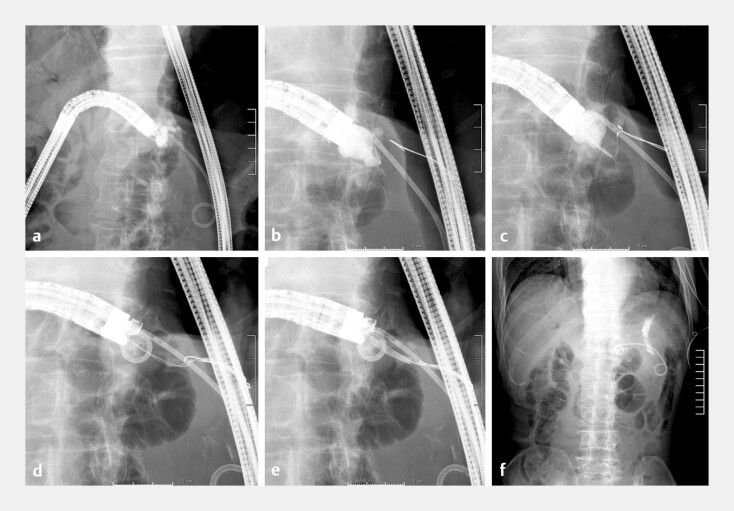
Endoscopic ultrasound-guided pancreaticojejunostomy. **a** Insertion of a forward-viewing echoendoscope into the pancreaticojejunostomy site, followed by pancreatic duct puncture using a 19G needle and contrast medium injection. **b** Guidewire placement in the pancreatic duct. **c** Puncture site dilation using a drill-type dilator. **d** Placement of two guidewires using a double-lumen catheter. **e** Puncture site dilation with a 3-mm balloon dilator. **f** Stent placement in the pancreatic duct. Abbreviation: EUS, endoscopic ultrasound.

Endoscopic ultrasound (EUS)-guided pancreaticojejunostomy after unsuccessful attempts with a balloon enteroscope and a transgastric EUS-guided approach.Video 1

Endoscopy_UCTN_Code_TTT_1AS_2AI

## Notes

Endoscopy E-Videos https://eref.thieme.de/e-videos
*E-Videos* is an open access online section of the journal *Endoscopy* , reporting on interesting cases and new techniques in gastroenterological endoscopy. All papers include a high-quality video and are published with a Creative Commons CC-BY license. Endoscopy E-Videos qualify for HINARI discounts and waivers and eligibility is automatically checked during the submission process. We grant 100% waivers to articles whose corresponding authors are based in Group A countries and 50% waivers to those who are based in Group B countries as classified by Research4Life (see: https://www.research4life.org/access/eligibility/ ). This section has its own submission website at https://mc.manuscriptcentral.com/e-videos .
